# Metta-Based Therapy for Chronic Depression: a Wait List Control Trial

**DOI:** 10.1007/s12671-021-01753-y

**Published:** 2021-10-08

**Authors:** Ulrich Stangier, Artjom Frick, Isabel Thinnes, Elisabeth A. Arens, Stefan G. Hofmann

**Affiliations:** 1 Clinical Psychology and Psychotherapy, Department of Psychology, Goethe University Frankfurt, Varrentrappstr. 40–42, 60486 Frankfurt am Main, Germany; 2 Department of Psychological and Brain Sciences, Boston University, Boston, USA

**Keywords:** Metta, Loving kindness, Mindfulness meditation, Cognitive behavioral therapy, Chronic depression, Persistent depressive disorder

## Abstract

**Objectives:**

Current treatments for chronic depression have focused on reducing interpersonal problems and negative affect, but paid little attention to promoting prosocial motivation and positive affect. Following this treatment focus, the objective of the present study was to examine whether the combination of metta (Loving Kindness) group meditation and subsequent tailored individual therapy focusing on kindness towards oneself and others (metta-based therapy, MBT) shows greater improvements in depressive symptoms than a wait list control group in patients with chronic depression.

**Methods:**

Forty-eight patients with DSM-5 persistent depressive disorder were randomly assigned to MBT or a wait list control condition. Outcome was assessed after group meditation, after subsequent individual therapy, and at 6-month follow-up. The primary outcome measure was an independent blind rating of depressive symptoms at post-test. Secondary outcome included changes in self-reported depression, behavioral activation, rumination, social functioning, mindfulness, compassion, and clinician-rated emotion regulation.

**Results:**

Mixed-design analyses showed significant differences between MBT and WLC in changes from pre- to post-test in clinician-rated and self-rated depression, behavioral activation, rumination, social functioning, mindfulness, and emotion regulation. Most of the changes occurred during group meditation and were associated with large effect sizes. Improvements were maintained at 6-month follow-up.

**Conclusions:**

The results provide preliminary support for the effectiveness of MBT in treating chronic depression.

**Trial Registration:**

ISRCTN, ISRCTN97264476.

Persistent depressive disorder (PDD)—or chronic depression—is a highly prevalent mental disorder, with a lifetime prevalence of 4.6% (Murphy & Byrne, 2012). In contrast to major depressive episodes, the condition is defined by at least mild symptoms of depression persisting for more than 2 years (American Psychiatric Association, 2013). Chronic depression is associated with poor psychosocial functioning (Rhebergen et al., 2009). As compared to non-chronic depression, comorbidity rates and risk of suicide are significantly increased (Gilmer et al., 2005). In general, outcome of pharmacological and psychological treatments is significantly lower than that for non-chronic forms of depression (Kriston et al., 2014).

Among the psychological factors that may contribute to the maintenance of a chronic course in depression, dysfunctional strategies of emotion regulation are of major importance. Chronic depression is associated with an increased tendency to avoid and suppress negative thoughts and emotions (Brockmeyer et al., 2015) and positive affect (Hofmann et al., 2012; Joormann & Stanton, 2016). In addition, ruminating about negative affect and the meanings of depressive symptoms contributes to the maintenance of depression (Nolen-Hoeksema, 2000). Moreover, ruminating about positive emotion prevents patients from upregulating their mood through positive emotions (Vanderlind et al., 2020).

Persistence of depression has also been explained by dysfunctional interpersonal patterns. In line with McCullough’s (2000) theory of impaired social cognitions, chronically depressed patients lack empathy towards others (Schnell & Herpertz, 2018). Finally, an increased frequency of childhood trauma and childhood adversity has been found in PDD, including emotional and sexual abuse as well as emotional neglect (Liu, 2017). Early adversity may create dysfunctional early maladaptive schemas, characterized by self-defeating cognitive emotional patterns regarding oneself and one’s personal relationships in chronic depression (Renner et al., 2012).

Current approaches of psychological treatments for chronic depression largely focus either on interpersonal problems, such as Cognitive Behavioral Analysis System of Psychotherapy (CBASP), or on emotion regulation, such as Mindfulness-Based Cognitive Therapy (MBCT). In a previous randomized controlled trial with chronically depressed patients, CBASP showed significantly larger effects than MBCT in clinical ratings, but not self-ratings, of depression (Michalak et al., 2015). In another trial, however, the effects of cognitive behavioral therapy emphasizing mindfulness exercises and behavioral activation did not differ significantly from CBASP in chronically depressed patients (Rief et al., 2018). Finally, also schema therapy showed promising results with large effect sizes in two single case series studies (Malogiannis et al., 2014; Renner et al., 2016).

Whereas most treatments for chronic depression focus on negative patterns of cognitions, emotion regulation, and interpersonal problems, there is also a need for promoting positive patterns of affect and interpersonal behavior (Hofmann, 2014). As an intervention that targets prosocial motivation and social connectedness, metta (Loving Kindness) meditation aims to increase the wish to promote the well-being of others and of the self (Hofmann et al., 2011). In Buddhism, *metta* (Pali; “benevolence,” “loving-kindness,” “kindness”) refers to a mental state of unselfish and unconditional kindness to all beings that one develops through meditation and cultivation in relations with others. Metta is to be distinguished from *karuna* (compassion) which focuses on the wish to reduce the suffering of others and the self (Gilbert, 2009). Metta meditation which usually builds upon mindfulness meditation has been repeatedly shown to enhance prosocial behavior, increase psychological well-being, improve interpersonal relationships, and reduce symptoms of depression in clinical and nonclinical samples (Galante et al., 2014). Furthermore, a recent study compared the effects of metta and mindfulness meditation in non-clinical individuals and found that only metta but not mindfulness meditation was associated with reduced social avoidance goals and increased social approach goals during the intervention (Don et al., 2021). According to the broaden and build theory by Fredrickson (2004), metta meditation can trigger upward spirals of experiencing positive affect, widening consciousness, flexible thinking, and increase of behavioral resources to improve interpersonal relationships and psychological well-being. In line with these findings, we found strong reductions in depressive symptoms after metta group meditation in two pilot studies with chronically depressed patients (Graser et al., 2016; Hofmann et al., 2015).

In the present study, we tested the efficacy of an 8-session group treatment integrating the principle of group meditation combined with 8 sessions of individual therapy. Group intervention comprised mindfulness and Loving Kindness meditation. The individual interventions focused on the activation of kind behavior and the modification of dysfunctional schemas. We tested the following hypotheses: Compared to the wait list control condition, the combination of group meditation and individual therapy (metta-based therapy, MBT) will show a greater reduction in depressive symptoms from baseline to post-assessment (Hypothesis 1). In addition, we investigated whether significant changes also occurred in secondary measures including behavioral activation, rumination, mindfulness, compassion, and emotion regulation (Hypothesis 2). We also predicted that these changes will be maintained over a 6-month follow-up (Hypothesis 3). Furthermore, we examined the role of mindfulness and compassion as mediators for treatment outcome (Hypothesis 4). Finally, we also explored whether changes in depressive symptoms and secondary outcomes occurred (a) from before to after the group part of therapy, as well as (b) from before to after the individual part of therapy.

## Methods

### Participants

Participants were recruited in the Frankfurt metropolitan region through the Center for Psychotherapy at the Goethe University Frankfurt, self-help groups, psychosocial counselling centers, flyers, and advertisements on websites. Inclusion criteria comprised (1) primary diagnosis of persistent depressive disorder according to DSM-5, including as the main criterion “depressed mood for most of the day, for more days than not, as indicated by either subjective account or observation by others, for at least 2 years” (see APA, 2013, p. 168, for the definition of criteria A–H); (2) age 18–70 years; (3) no current psychotherapeutic treatment; (4) written consent to participate in the study. Diagnoses were assessed by trained, independent assessors, using the German version of the SCID adapted to DSM-5 (Falkai & Wittchen, 2015) and the Psychiatric Status Rating (Keller et al., 1987), adapted for chronic depression, to obtain more reliable assessments of the diagnostic criteria related to severity and chronic course of symptoms (criteria A-E and H, APA, 2013, p. 168). Exclusion criteria were as follows: (1) acute suicidality, (2) substance abuse or dependence syndrome within the past 3 months, (3) psychotic disorders, (4) bipolar disorder, (5) borderline personality disorder, (6) organic mental disorder, or (7) serious physical illness. Concurrent psycho-pharmacological treatment was not an exclusion criterion. Patients continued to receive a pharmacological anti-depressant treatment if indicated and were encouraged to keep it constant. Changes in medication were recorded and documented.

Based on within-group effect sizes from previous pilot studies (Graser et al., 2016; Hofmann et al., 2015), we assumed at least a moderate effect of *f* = 0.25 in comparison to the wait list control group. A power analysis was computed using G-Power, with repeated measures ANOVA (within-between interaction), a power of 0.80, and a correlation among the repeatedly measured dimensions of *r* = 0.7, resulting in a sample size of 34. Accounting for an estimated drop-out rate of 25%, and to achieve balanced group sizes, we determined the sample size to be 48 patients.

### Procedures

#### Trial Design

We employed a single-center, block randomization, parallel-group (MBT versus wait list control condition) design (Frick et al., 2020). Since childhood trauma influences the course of illness and treatment outcome in depression (Nanni et al., 2012), the sample was stratified based on the level (high vs. low) of childhood trauma as measured by the Childhood Trauma Questionnaire (CTQ; Bernstein et al., 2003) which was completed in the eligibility screening. Participants in the control group received no treatment or treatment as usual (e.g., antidepressants), but no psychotherapy during the treatment of the experimental group. The primary outcome measure was clinician-rated symptoms of depression, rated by blinded independent assessors at four time points: before intervention (T0); after group meditation (T1); after individual therapy (T2); and at 6-month follow-up (T3). T3 was assessed only in the treatment group because the wait list condition terminated after T2 and was offered MBT. The study protocol was approved by the Department of Psychology’s Research Ethics Committee at Goethe University Frankfurt and registered with ISRCTN (ISRCTN97264476).

Out of 135 individuals who had registered their interest to participate and had been pre-assessed in a brief telephone screening, 79 participants were invited for a clinical interview conducted by trained, independent clinicians to assess inclusion criteria. Based on the German version of the SCID and the Psychiatric Status Rating adapted for Chronic Depression, 48 participants were eligible for inclusion and randomized into one of two conditions, treatment or wait list control. Randomization was performed by an individual external to the current study through computer-generated random lists. Two individuals chose not to participate after the random allocation and before baseline assessment took place, and were replaced by other individuals meeting the inclusion criteria.

**Fig. 1:**
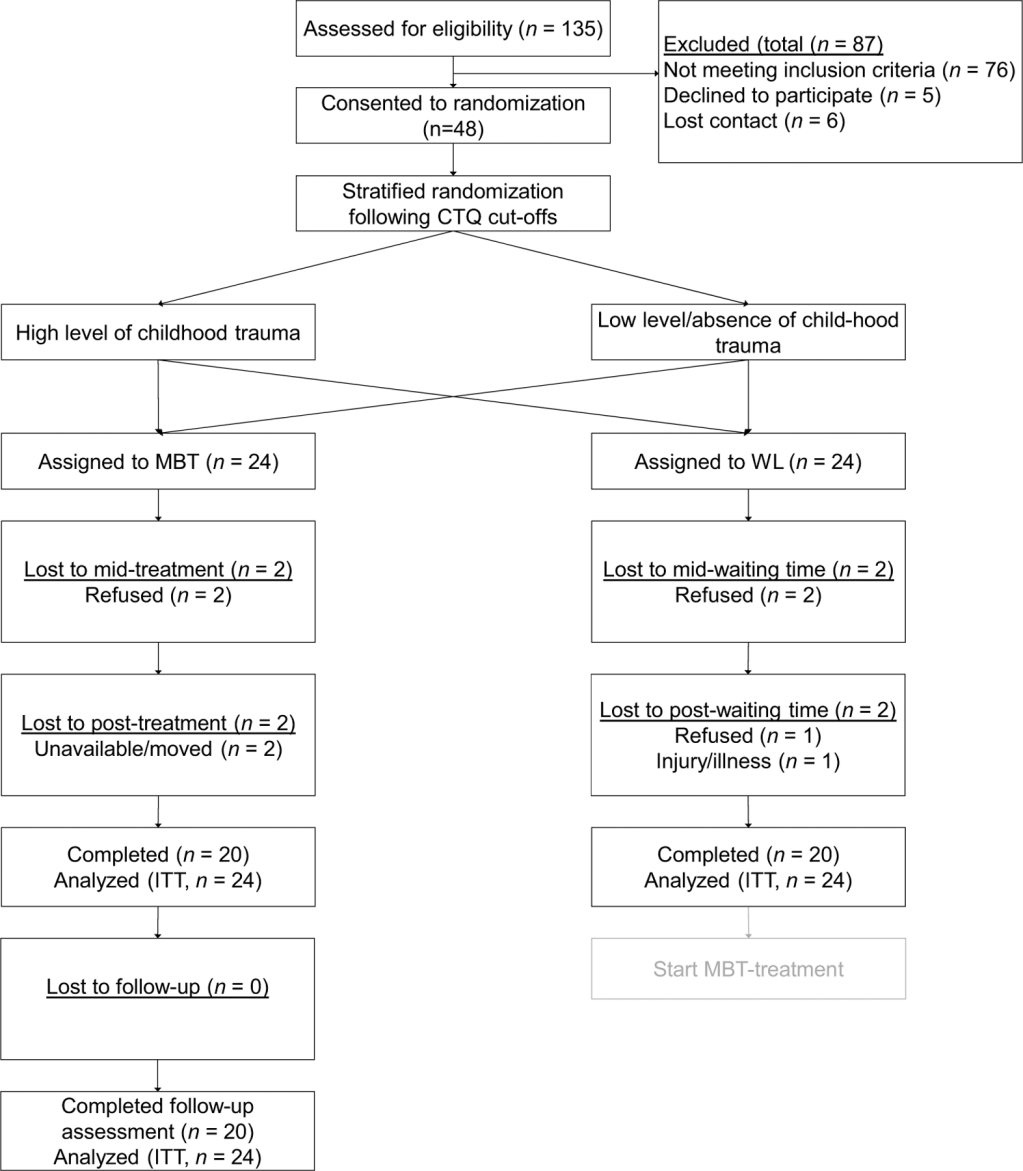
Flowchart of subjects. MBT, metta-based therapy; ITT, intention-to-treat; WL, wait list control group CTQ, Childhood Trauma Questionnaire

The experimental and wait list control groups were analyzed on primary and secondary outcome measures and sociodemographic variables at pre-treatment assessment to check for significant group differences. Chi-square tests and Student’s *t*-tests for independent samples indicated that there were no significant baseline differences between the groups (see Table 1). The subject flow diagram is shown in Fig. 1.

#### Treatment

The 4-month manualized treatment program combined group meditation, provided for twelve participants, with individual therapy (8 sessions, 100 min). The treatment focused on motivation for kindness towards oneself and others (Table 2). The group meditation program comprised 8 sessions (100 min) and one half-day retreat (4 h) and consisted of exercises focusing on mindful meditation (body scan, sitting meditation, breathing space, walking meditation [Segal et al., 2013]) and loving kindness meditation, as proved in the pilot studies (Graser et al., 2016; Hofmann et al., 2015). Metta meditation was based on a short mindfulness introduction and consisted of silent repetitions of phrases such as “may you be happy” directed at oneself, a friend, a neutral person, a “difficult” person, all four together, and all human beings (Hofmann et al., 2011). Emphasis was put on daily homework practice. In addition, we included information about philosophic foundations of metta in Buddhism, benevolence in ancient and modern Western philosophy, and psychological and neurobiological research on kindness, as well as structured reflection exercises on the importance of this attitude for personal well-being (Arieli et al., 2014).

The individual therapy comprised 8 sessions of 100 min and focused on the implementation of kindness into daily life. Treatment goals were derived from functional analyses (Hofmann & Hayes, 2019) focusing on self-critical or hostile cognitive schemata. The interventions used to increase kind attitudes and behaviors included the continuation of meditation practice and CBT techniques. Using behavioral activation (Martell et al., 2010), patients were encouraged to increase behaviors related to self-kindness and kindness towards others (Mongrain et al., 2018; Nelson et al., 2016), and to identify dysfunctional cognitions preventing them from kindness. In case the dysfunctional pattern was related to childhood maltreatment, empty chair dialogue and imagery rescripting were also used to identify and modify maladaptive schemas (Renner et al., 2013).

Both treatment components were carried out by four clinical psychologists who were at an advanced stage or had completed a post-graduate training in cognitive-behavioral therapy. The group therapists were trained in mindfulness-based interventions and MBT, had received supervision by an experienced mindfulness teacher (Dr. Thomas Heidenreich), and had participated in the pilot studies on metta meditation. All therapists had conducted individual pilot treatments and received biweekly supervision focusing on the adherence to the mindfulness-based treatment manual (Stangier et al., 2021).

### Measures

Primary outcome measure was the clinician-rated severity of depressive symptoms as measured by the Quick Inventory of Depressive Symptomatology (QIDS-C; Rush et al., 2003). The QIDS-C consists of sixteen items, scored according to severity on a 0–3 scale assessing the DSM diagnostic criteria for depression. The total score ranging from 0 to Cronbach’s alpha for this scale with the current sample was 0.62 which is slightly below values reported in other studies (Reilly et al., 2015). On the basis of 12 randomly selected interviews with patients with chronic depression (*n* = 12) and other diagnoses, an interrater reliability of *r* = 0.97 was achieved.

Secondary outcome measures included the following self-rating instruments: (1) the Beck Depression Inventory (BDI-II; Beck et al., 1996), which contains 21 items referring to symptoms of depression experienced during the past week. The total score ranges from 0 to 63. For the total scale, Cronbach’s alpha in the current study was 0.86. (2) The Behavioral Activation for Depression Scale (BADS; Kanter et al., 2007). The BADS is a 25-item self-report scale comprising four subscales measuring activation, avoidance, and rumination as well as related impairments in work and social life. In the current study, Cronbach’s alpha of the total scale demonstrated good internal consistency (*α* = 0.86). (3) The Compassionate Love Scale (CLS; Sprecher & Fehr, 2005), which is a 21-item self-report measure that evaluates the degree to which one feels compassion or altruistic love towards others, selfless caring, and the motivation to help. The CLS exists in two versions: (a) compassion toward close others (friends, family) and (b) compassion toward strangers or all humanity. In the present study, a mean score was calculated from both versions. Items were rated on a 7-point Likert-type scale (1 = not at all true of me; 7 = very true of me). In the current sample, Cronbach’s *α* was 0.96. (4) the Five Facet Mindfulness Questionnaire (FFMQ; Baer et al., 2006). The FFMQ is a 39-item questionnaire measuring self-directed mindfulness by five factors: “Observing,” “Describing,” “Acting with attention,” “Accepting without judgment,” and “Non-reactivity.” Cronbach’s *α* of the total score in the current sample was 0.83. (5) The Response Styles Questionnaire (RSQ-D; Nolen-Hoeksema, 1991). To assess persistent tendency to rumination, the RSQ-D was used. The questionnaire consists of 32 items measuring the two coping styles rumination and distraction when dealing with depressive mood. Internal consistency of the rumination subscale was 0.75. (6) The Social Adaptation Self-evaluation Scale (SASS; Bosc et al., 1997). The SASS is a 21-item scale for the evaluation of social functioning in different areas, including work, spare time, family, environmental organization, and coping abilities. Each item is rated on a four-point scale. Cronbach’s alpha for the total scale was acceptable with *α* = 0.78.

All measures were completed at T0 – T2 in both study arms, and at T3 in the treatment condition only. Due to a mistake in the implementation of the study protocol (Frick et al., 2020), the social pain questionnaire was confused with another questionnaire. Thus, the social pain questionnaire was only collected in about half of the participants and excluded from data analyses. The results of the remaining measures included in the study protocol may be reported in a separate future paper when appropriate.

In addition, blind and trained raters assessed emotion regulation skills using the subscales of the Interview for Operationalized Skills Assessment (German version: OFD; Stenzel et al., 2010) at pre- and post-treatment. This semi-structured interview assesses the adaptiveness of emotion regulation on five dimensions (acceptance of emotions, impulse control and purposeful behavior, identification and naming of emotions, expression of emotions, and access to strategies for emotion regulation) associated with negative emotions in different areas of life. Based on 12 interviews with patients with chronic depression and other diagnoses, an interrater reliability of *r* = 0.97 was observed.

### Data Analyses

The results are reported on the basis of intent-to-treat analysis. To account for missing data, multiple imputation was performed in *R* (version 4.0.3) using the MissForest package (version 4.6–14). The procedure, an iterative imputation method based on a random forest, utilized all of the primary and secondary outcome measures at item level. The maximum number of iterations was set to 10 (maxiter = 10), and the number of regression trees for each iteration was set to 1000 (ntree = 1000).

Sample characteristics of treatment group and wait list control group were compared by univariate ANOVAs or *χ*^2^ tests. A mixed-design (three-level factor Time by two-level factor Group by two-level CTQ-based stratifier Childhood Trauma with “no childhood trauma” vs. “at least one childhood trauma”) analysis of variance (ANOVA) was performed on the primary outcome measure to investigate the treatment results at post-treatment assessment as reflected by Group × Time interaction effects. A mixed-design MANOVA using Pillai’s Trace followed by univariate analyses was calculated to test Group by Time interaction effects for secondary measures at post-treatment. The significance level for the univariate ANOVAs of the secondary outcome measures was Bonferroni-adjusted by dividing the *p*-value by the number of outcome variables. Thus, the significance level of *p* = 0.05 and seven secondary outcome measures was Bonferroni-adjusted to a significance level of *p* = 0.007.

Additional exploratory analyses including midterm assessment (after group treatment) and follow-ups were performed using post hoc contrasts to examine changes in depression after group and individual treatments. All calculations were conducted using SPSS 27.

Controlled effect sizes were calculated using *d*_*ppc2*_ (pre-test–posttest-control design), with the difference in the pre-post changes between treatment and wait list control conditions, divided by the pooled pretest standard deviation, and a bias correction (Morris, 2008). To calculate the effect size from post-treatment to 6-month follow-up in the treatment group, we calculated *d*_*RM*_ using the sample standard deviation of the mean difference adjusted by the correlation between measures.

Treatment response was defined as a 50% or greater reduction in the baseline QIDS-C by the end of the treatment and follow-up (Rush et al., 2006). Remission was determined by a threshold of ≤ 5 based on the QIDS-C as recommended by Trivedi et al. (2004). To allow comparisons with previous trials, response and remission rates were additionally determined on the basis of the BDI-II, with remission defined as BDI-II ≤ 13 (Beck et al., 1996), and response as a decrease of 50% from baseline (Reeves et al., 2012).

To examine clinically significant improvement/deterioration, we used criteria of Jacobson and Truax (1991) to compute reliable change indices (RCI) in the QIDS-C and BDI-II. Significant improvement was determined by scores exceeding 1.96. Deterioration was determined using a negative change score exceeding the RCI, as recommended by Jacobson and Truax (1991).

## Results

### Attrition, Adherence, and Changes in Medication

Twenty participants (83%) assigned to treatment completed all treatment sessions (see Fig. 1). Four participants (17%) withdrew from both the treatment and the wait list control groups. Drop-out was defined as not completing the post-treatment assessment regardless of the number of completed treatment sessions. Independent samples *t*-tests did not reveal a difference in terms of completion for outcome measures and sociodemographic variables. Results of Little’s MCAR-test indicated that data were missing at random, *χ*^2^(24,166, *N* = 48) = 1760.9, *p* > 0.999.

Based on completer data at post-treatment, 55% did not change medication, 20% discontinued medication, 15% reduced the medication dose, and 10% increased the dose in the treatment group. In the wait list control group, 85% showed no change in medication, 5% discontinued, 5% reduced, and 5% increased their dose. There was no significant difference between the two groups, *χ*^2^(3, *N* = 40) = 4.4, *p* = 0.220). Two out of four drop-outs in the treatment group had received medication, as did two out of four dropouts in the control group.

### Treatment Effects

**Fig. 2:**
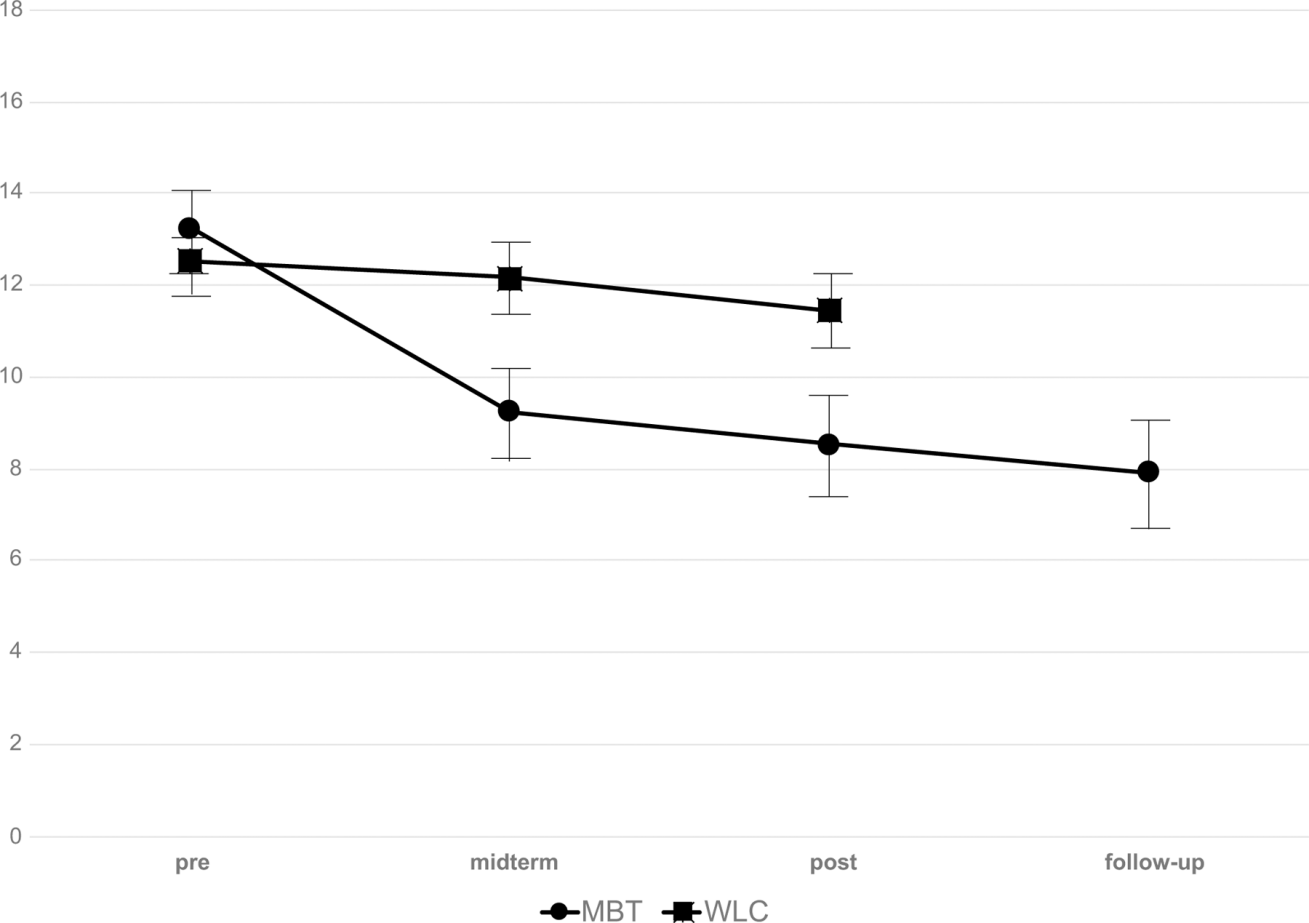
Depressive symptom severity (QIDS) from baseline to week 8 (mid-treatment) and to week 16 (post-treatment) by treatment group. Values are means (with SEM) from intention-to-treat analysis in metta-based cognitive behavioral therapy (MBT) and wait list (WL)

The descriptive statistics for the primary and secondary outcome measures can be obtained from Table 3. A mixed-design ANOVA on the primary outcome measure (QIDS-C, Hypothesis 1) showed a significant Group × Time interaction, *F*_(1,46)_ = 6.21, *p* = 0.016, indicating improvement in the clinician-rated depression at post-treatment in the treatment in MBT, as compared to the wait list control group (see Table 4 and Fig. 2). No significant interaction effect on primary outcome was found for Time × Childhood trauma, *F*_(2, 88)_ = 0.44, *p* = 0.641, or Group × Time × Childhood trauma, *F*_(2, 88)_ = 0.30, *p* = 0.743. Since no significant interaction effects for childhood trauma occurred in any of the secondary outcome variables, and no difference was found between effects when including or omitting the interaction with childhood trauma, the following results are only reported for Group × Time interaction to ensure clarity of the presentation.

A mixed-design MANOVA on secondary outcome measures (Hypothesis 2) using Pillai’s Trace showed a significant interaction effect of Group × Time, *F*_(10, 37)_ = 2.94, *p* = 0.008. Subsequent univariate analyses revealed significant Group × Time effects for depression (BDI-II), behavioral activation (BADS), mindfulness (FFMQ), and rumination (RSQ), but not for compassion (CLS) (test statistics see Table 4). The completer analysis obtained similar results.

For the primary outcome, there was no significant interaction effect of Time × Antidepressant Medication, *F*_(2, 84)_ = 0.17, *p* = 0.891. However, the interaction effect of Group × Time × Antidepressant Medication was significant, *F*_(2, 88)_ = 5.03, *p* = 0.009. There was a larger difference in favor of the treatment in those patients who did not receive medication (MBT: *M*_pre_ = 13.78, *SD* = 4.41, *M*_post_ = 6.61, *SD*_post_ = 5.52; WLC: *M*_pre_ = 11.36, *SD*_pre_ = 2.37, *M*_post_ = 11.84, *SD*_post_ = 4.46), than in those patients who received medication (MBT: *M*_pre_ = 12.93, *SD* = 4.42, *M*_post_ = 9.71, *SD*_post_ = 4.73; WLC: *M*_pre_ = 14.20, *SD*_pre_ = 3.61; *M*_post_ = 10.90, *SD*_post_ = 2.19). A mixed-design MANOVA including all secondary outcome measures with the within-subjects factor Time (pre-post) and the two-level between-subjects factors Group and Medication (intake vs. no intake) showed no significant interaction effect of Time × Antidepressant Medication, *F*_(7, 38)_ = 1.61, *p* = 0.162, and Time × Group × Antidepressant Medication, *F*_(7, 38)_ = 1.62, *p* = 0.159.

To explore the changes in the two stages of the treatment and from post-assessment to follow-up, we calculated mixed-design ANOVAs comparing the differences in changes between baseline and mid-treatment (after group meditation); mid-treatment and post-treatment (after individual therapy); and within-group effects from post-treatment to 6-month follow-up. The differences between baseline and mid-treatment were significant for QIDS-C, BDI-II, the BADS total score, and the FFMQ total score (Table 4). Time by treatment effects increased significantly from mid-treatment and post-treatment only in terms of mindfulness and symptom-related rumination. From post-treatment to 6-month follow-up (Hypothesis 3; assessed only in the experimental group), positive effects were maintained in all outcome variables (Table 4).

### Preliminary Mediation and Moderation Tests

A mediation test (Hypothesis 4) showed that pre to post changes in mindfulness (FFMQ) significantly mediated the effect of the intervention on the pre to post change of depressive symptoms (QIDS) (standardized indirect effect = 0.201, *p* = 0.024). A reverse mediation test showed that the pre-post reduction of depressive symptoms did not mediate the intervention effect on the pre-post change in mindfulness (− 0.116, *p* = 0.063). The direct effect without the mediator (standardized effect = − 0.506, *p* < 0.001) did not change substantially through inclusion of the mediator (standardized effect = − 0.390, *p* = 0.002). These results suggest that the therapy effect was mediated through the change in mindfulness.

Originally intended corresponding mediation analyses with the CLS were not conducted since the CLS showed no significant change over the course of treatment. However, we conducted a moderation test to explore whether the baseline levels of CLS moderated the effect of treatment on depressive symptoms. We found a trend for the change from mid-to post-treatment (standardized interaction effect = 1.49, *p* = 0.056), indicating that in the treatment group, a high baseline CLS value tended to predict a stronger reduction in depressive symptoms in the second treatment half. No interaction was found for the pre-mid-treatment phase (standardized interaction effect = − 0.396, *p* = 0.615), and the pre-post measurement (standardized interaction effect = 0.919, *p* = 0.212).

### Response, Remission, Clinically Significant Improvement and Deterioration

At the end of the treatment, rates of treatment response and remission based on QIDS and BDI-II scores were significantly higher in the MBT group than those in the control group (Table 5). At follow-up, about half of the participants in the treatment group met criteria for response and remission. Clinically significant improvement from baseline to post-assessment occurred in 54.2% of the treatment group, based on the QIDS. Based on BDI-II, the rate was 75%. Clinically significant deterioration scores were low overall and did not differ between groups, neither when based on QIDS nor on BDI-II scores (Table 5).

## Discussion

The aim of the current study was to evaluate the efficacy of the metta-based therapy, a combination of group meditation and individual therapy aiming to increase a kind attitude and behaviors related to oneself and others. The program was proven highly effective in reducing depressive symptoms, rumination, and cognitive and behavioral avoidance, as well as improving social adaptation, emotion regulation, and mindfulness. Effects of treatment turned out to be stable at a 6-month FU, indicating the long-term efficacy. The results confirm the promising outcome of uncontrolled pilot studies (Graser et al., 2016; Hofmann et al., 2015) and expand it as the treatment was superior to a symptom reduction caused by the passage of time, expectation, or testing. The findings are substantial in light of the long history of depression and unsuccessful applications of various treatments for the majority of the participants.

For the primary outcome measure, we found significant and large effects of clinical ratings of depression in favor of the treatment program as compared to wait list control. The effect size (*d* = 0.93) is higher than effect sizes obtained in previous studies for MBCT (*d* = 0.29) and comparable to CBASP (*d* = 0.85) in the study by Michalak et al. (2015). In addition, also the rates for response (37.5%) and remission (25%), although on a low level, were comparable to previous findings for CBASP (Schramm et al., 2017) and higher than for MBCT (Michalak et al., 2015). However, a direct comparison to previous studies is biased since we used a wait list control condition. Although half of the participants in our study received medication and were in psychiatric treatment, which is comparable to treatment as usual conditions in the study by Michalak et al. (2015), reliable evidence can only be provided by a direct comparison of treatments in a randomized controlled trial.

Interestingly, the effects of MBT on self-reported symptoms, as assessed by the BDI-II, were even higher level than for the clinician-rated QIDS (*d* = 1.46 vs. 0.93). The lower sensitivity of clinician ratings contrasts with previous studies (Carrozzino et al., 2020), but may be explained by the complementary focus of symptoms assessed in both modalities. Whereas the BDI and other self-report measures emphasize cognitive and emotional symptoms such as rumination and despair, clinician ratings rather focus on behavioral and somatic symptoms (Uher et al., 2012). This may also explain the higher rate of clinically significant changes based on the BDI-II (75%), as compared to the QIDS (54.2%).

Concomitant medication was associated with a reduced efficacy and even appeared to neutralize the effects of MBT. Although this finding conflicts with a recent meta-analysis (Cuijpers et al., 2019), a recent systematic review (Whiston et al., 2019) found that the outcome of CBT was better without concomitant medication. A possible explanation is that the use of antidepressants may be associated with emotional blunting (Goodwin et al., 2017), which counteracts the effects of mindfulness and metta meditation as well as interventions targeting cognitive and emotional processes.

A potential mediator of treatment outcome is the improvement of mindfulness, accompanied by reduced rumination, which is in line with the evidence from reviews indicating that increased mindfulness and decreased rumination mediate the effects of MBCT on depression (Van der Velden et al., 2015). Furthermore, the large effect of MBT on the independent clinical rating of emotion regulation in our study indicates that the treatment also improved the abilities to identify, accept, and express negative emotions, to cope with emotional distress, and to maintain behavioral control.

In contrast to our expectation, we did not observe significant changes in compassion, although individuals high in compassion at baseline tended to benefit more from treatment. Another possible explanation for the absence of significant changes may be that high baseline levels and a ceiling effect might have prevented the detection of any treatment effects. Furthermore, the CLS may not precisely measure the target of our program, since compassion focuses on the suffering of others, but kindness on the well-being and happiness (Gilbert et al., 2019). Unfortunately, there is no validated instrument that refers specifically to kindness (Strauss et al., 2016).

Although the large effects in the behavioral activation scale indicate that participants strongly increased their engagement in activities in general, we focused the interventions on benevolent activities towards oneself and others (Mongrain et al., 2018; Nelson et al., 2016). By increasing prosocial motivation and reducing self-criticism, metta meditation may help chronically depressed patients to overcome lack of interest and social withdrawal (Stefan & Hofmann, 2019). According to Fredrickson’s broaden and build model of positive emotions, metta meditation triggers a spiral of positive emotions and personal resources, including the ability to savor positive experiences and the improvement of relations with others and social support (Fredrickson et al., 2008). This upward spiral may also explain the significant, although moderate, increase in social adjustment in the treatment group.

The main proportion of reduction in depressive mood and associated depressive symptoms was achieved after group meditation. However, substantial gains in behavioral activation, mindfulness, and the reduction of rumination were made during subsequent individual therapy. Thus, additional changes in cognitive processing occurred during individual therapy, which may have also stabilized the benefits of the preceding metta group meditation until follow-up.

### Limitations and Future Research

Despite the promising results, our study suffers from several limitations. An important limitation is the use of a wait list control group. Although this allows for the control of passage of time (such as regression to the mean and seasonal changes) and confounding factors, no conclusion can be drawn with comparison to active psychological treatments. Furthermore, since the group and individual treatment elements were presented in a fixed sequence, we will not be able to determine the specific influences of the two treatment components on the overall outcome. Third, we did not apply a formal testing of treatment fidelity. Since the structure of the individual therapy was largely based on personalized functional analyses, the therapists were allowed to apply a broad arrangement of techniques focusing on kindness. Further studies are needed to operationalize behavioral criteria for the adherence and competence of the specific components of MBT, comparable to compassion-focused therapy (Horwood et al., 2020). Fourth, a strong allegiance with the treatment approach may have contributed to the large effects observed in this study. Therefore, we recommend that these findings be replicated in a large multicenter study controlling for treatment allegiance and other factors. Fifth, due to organizational reasons, participants were aware of their allocation before baseline assessment. Thus, knowing their allocation may have motivated participants to report better or worse scores in the baseline outcome measures. Another limitation is that multiple constructs were measured using multiple‐item scales presented within the same survey, which could lead to spurious effects due to the measurement instruments rather than to the constructs being measured (Podsakoff et al., 2012). Finally, the follow-up interval of 6 months is not appropriate to assess long-term changes in chronic depression. Enduring effects of interventions may be demonstrated by a 1- or 2-year follow-up.

Our findings suggest that MBT is an effective intervention for depression, and possibly other conditions associated with self-criticism and social impairments (Johnson & Wood, 2017). These findings justify a large-scale multicenter trial to support the efficacy of combining group meditation and individual therapy focusing on kindness.

## Figures and Tables

**Table 1:** Sample characteristics

	MBT	Waiting-list control	*p*
Age (mean +—SD), years	51.58 (11.26)	48.92 (11.39)	.419^a^
Female, *n* (%)	66.67	83.33	.182^b^
Upper secondary education, *n* (%)	82.61	80.95 (17 of 21)	
Age of onset (mean ± SD)	21.75 (11.77)	22.70 (12.97)	.794^a^
QIDS-C at baseline (mean ± SD)	13.25 (4.34)	12.54 (3.22)	.524^a^
Childhood Trauma Questionnaire: at least one trauma, *n* (%)	13 (54.2%)	15 (62.5%)	.558^b^
Antidepressant medication, *n* (%)	15 (62.5%)	9 (40.9%)	.143^b^

a By analysis of variance

b By *χ*^2^ test

**Table 2:** Overview of metta-based therapy: goals and techniques

Setting	Goals / modules	Techniques
Group		
4 sessions (weekly)	Increasing mindfulness	Meditation: breathing space, body scan, sitting meditation; daily practices (audiotaped and self-directed)
5 sessions (weekly)	Increasing kindness	Meditation: Cultivate wishing good to self and others (i.e., wishing happiness, safety, health, well-being) by daily practices of metta meditation (audiotaped and self-directed) Dyadic group exercises: specifying kind wishes towards self and others, identifying barriers Homework reflection on kindness/benevolence toward self and others; reading handouts, writing essays
Individual		
1 session	Functional analysis	Deriving a model relating triggers of depressed mood to processing (e.g., rumination), thoughts (e.g., self-criticism), and behaviors (e.g., withdrawal), and a positive model including kindness towards self and others
	Setting individual goals	Specification of personal values, barriers, and resources related to kindness; analysis of adverse past experiences
6 sessions (biweekly)	Increasing kindness	Continuation of individualized mediation practice (in-session and homework)
	Turning kindness into action	Scheduling activities of doing good to self and others (i.e., engaging in daily acts of kindness)
	Overcoming barriers to kindness	Mindful distancing from rumination Cognitive restructuring and behavioral experiment to test dysfunctional thoughts related to kindness Imagery rescripting and chair dialogue in case of adverse past experiences
1 session	Relapse prevention	Closing balance of gains and future tasks, recording of beneficial strategies, commitment to personal values

**Table 3:** Means and standard deviations of outcomes at baseline, after group treatment, at post-treatment, and at 6-month follow-up

	MBT *N* = 24	Waiting-list control *N* = 24
QIDS-C		
Baseline	13.25 (4.34)	12.54 (3.22)
After group treatment	9.25 (4.87)	12.17 (3.53)
Post-treatment	8.55 (5.15)	11.45 (3.65)
6-month follow-up	7.92 (5.60)	
BDI-II		
Baseline	32.19 (9.59)	27.71 (8.19)
After group treatment	19.12 (11.28)	25.20 (10.45)
Post-treatment	15.39 (10.21)	24.14 (8.98)
6-month follow-up	15.37 (11.90)	
BADS total score		
Baseline	56.42 (21.20)	68.97 (20.24)
After group treatment	80.17 (27.49)	72.24 (18.65)
Post-treatment	87.73 (29.08)	70.32 (20.64)
6-month follow-up	81.04 (22.60)	
CLS total score		
Baseline	4.66 (1.01)	4.98 (1.00)
After group treatment	4.70 (0.10)	4.90 (1.09)
Post-treatment	4.75 (1.05)	4.68 (1.06)
6-month follow-up	4.72 (1.09)	
FFMQ total score		
Baseline	107.75 (11.85)	108.76 (17.61)
After group treatment	117.45 (14.97)	109.58 (15.18)
Post-treatment	125.07 (18.06)	108.48 (18.79)
6-month follow-up	123.99 (17.60)	
RSQ total score		
Baseline	22.13 (4.22)	23.17 (4.06)
After group treatment	20.77 (4.13)	22.59 (3.21)
Post-treatment	17.90 (4.55)	23.32 (3.65)
6-month follow-up	18.06 (3.87)	
SASS total score		
Baseline	29.36 (5.51)	31.84 (7.72)
Post-treatment	35.17 (6.67)	33.44 (7.64)
6-month follow-up	34.15 (6.14)	
OFD emotion regulation total score		
Baseline	54.01 (8.77)	61.25 (15.62)
Post-treatment	71.01 (10.92)	57.13 (12.45)

**Table 4:** Test statistics and effect sizes at different stages of treatment (*after group treatment*, mid-treatment, at post-treatment, and at 6-month follow-up)

Outcome	*F*	*p* value	Effect size *d*
QIDS-C			
Group × time baseline to post-treatment	6.217	.016	− 0.93
Group × time baseline to mid-treatment	7.815	.008	− 0.94
Group × time mid-treatment to post-treatment	0.001	.982	–-
Time post-treatment to 6-month follow-up	0.232	.635	–-
BDI-II			
Group × time baseline to post-treatment	21.083	.000	− 1.46
Group × time baseline to mid-treatment	13.648	.001	− 1.16
Group × time mid-treatment to post-treatment	0.826	.368	–-
Time post-treatment to 6-month follow-up	0.495	.489	–-
BADS total score			
Group × time baseline to post-treatment	14.723	.000	1.42
Group × time baseline to mid-treatment	9.031	.004	0.97
Group × time mid-treatment to post-treatment	1.754	.192	–-
Time post-treatment to 6-month follow-up	2.666	.116	–-
CLS total score			
Group × time baseline to post-treatment	3.144	.083	–-
Group × time baseline to mid-treatment	0.399	.531	–-
Group × time mid-treatment to post-treatment	2.414	.127	–-
Time post-treatment to 6-month follow-up	0.366	.551	–-
FFMQ total score			
Group × time baseline to post-treatment	15.802	.000	1.15
Group × time baseline to mid-treatment	6.208	.016	0.58
Group × time mid-treatment to post-treatment	4.869	.032	0.57
Time post-treatment to 6-month follow-up	0.690	.415	–-
RSQ_SYM			
Group × time baseline to post-treatment	13.180	.001	− 1.04
Group × time baseline to mid-treatment	0.533	.469	–-
Group × time mid-treatment to post-treatment	9.594	.003	− 0.96
Time post-treatment to 6-month follow-up	1.740	.200	–-
SASS			
Group × time baseline to post-treatment	8.900	.005	0.62
Time post-treatment to 6-month follow-up	1.228	.279	–-
OFD			
Group × time baseline to post-treatment	56.920	.000	1.64

Degrees of freedom (*df*) of all baseline to post- and baseline to mid-treatment analyses as well as of all mid- to post-treatment analyses were *df* = 1,46. Df of all posttreatment to follow-up analyses were df = 1,23. –- refers to effect sizes smaller than moderate

**Table 5:** Response/remission rates, clinically significant improvement/deterioration rates

		MBT	WLC	
		n (%)	n (%)	χ^2^ (df = 1)
QIDS at post	Response	9 (37.5)	2 (8.3)	*χ*^2^ = 5.779, *p* < 0.05
	Remission	6 (25.0)	1 (4.2)	*χ*^2^ = 4.181, *p* < .05
	*Clinically significant improvement*	13 (54.2)	4 (16.7)	*χ*^2^ = 7.371, *p* < .01
	*Clinically significant deterioration*	1 (4.2)	2 (8.3)	*χ*^2^ = 0.356, *p* = .551
BDI-II at post	Response	11 (45.8)	2 (8.3)	*χ*^2^ = 8.545, *p* < .01
	Remission	8 (33.3)	3 (12.5)	*χ*^2^ = 2.948, *p* = .168
	*Clinically significant improvement*	18 (75)	7 (29.2)	*χ*^2^ = 10.101, *p* < .01
	*Clinically significant deterioration*	0	1 (4.2)	*χ*^2^ = 1.021, *p* = .312
QIDS at FU	Response	12 (50)		
	Remission	10 (41.7)		
BDI-II at FU	Response	13 (54.2)		
	Remission	12 (50)		

## Data Availability

All data are available at the Open Science Framework (https://osf.io/erswt/).
